# *Macleaya cordata* isoquinoline alkaloids attenuate *Escherichia coli* lipopolysaccharide-induced intestinal epithelium injury in broiler chickens by co-regulating the TLR4/MyD88/NF-κB and Nrf2 signaling pathways

**DOI:** 10.3389/fimmu.2023.1335359

**Published:** 2024-01-17

**Authors:** Yang Liu, Kai Han, Hua Liu, Gang Jia, Luke Comer, Guanlin Wang, Zizhu Pan, Yiqian Zhao, Shuzhen Jiang, Ning Jiao, Libo Huang, Weiren Yang, Yang Li

**Affiliations:** ^1^ Key Laboratory of Efficient Utilization of Non-grain Feed Resources (Co-construction by Ministry and Province), Ministry of Agriculture and Rural Affairs, Department of Animal Science and Veterinary Medicine, Shandong Agricultural University, Tai’an, China; ^2^ Institute of Animal Nutrition, Key Laboratory for Animal Disease-Resistance Nutrition of China, Ministry of Education, Sichuan Agricultural University, Chengdu, Sichuan, China; ^3^ College of Animal Science and Technology, Hunan Agriculture University, Changsha, China; ^4^ The Nutrition and Animal Microbiota Ecosystems Laboratory, Division of Animal and Human Health Engineering, Department of Biosystems, KU Leuven, Heverlee, Belgium

**Keywords:** broiler, Macleaya cordata isoquinoline alkaloid, intestinal epithelium injury, lipopolysaccharide, intestinal inflammation, oxidative stress

## Abstract

This study sought to explore the effects and potential mechanisms of dietary supplementation with isoquinoline alkaloids (IA) from *Macleaya cordata* to alleviate lipopolysaccharide (LPS)-induced intestinal epithelium injury in broilers. A total of 486 1-day-old broilers were assigned at random to a control (CON) group, LPS group, and LPS+IA group in a 21-d study. The CON and LPS groups received a basal diet, while the LPS+IA group received a basal diet supplemented with 0.6 mg/kg IA. At 17, 19, and 21 days of age, the LPS and LPS+BP groups were injected intraperitoneally with LPS, and the CON group was intraperitoneally injected equivalent amount of saline solution. The results manifested that LPS injection caused intestinal inflammation and lipid peroxidation, disrupted intestinal barrier and function, and increased the abundance of harmful microorganisms. However, dietary IA supplementation alleviated LPS-induced adverse changes in intestinal morphology, apoptosis, mucosal barrier integrity, cecum microorganisms, and homeostasis disorder by decreasing inflammatory cytokines and enhancing antioxidant-related genes expressions; inhibited LPS-induced increases in *TLR4* and *NF-κB* expressions and decreases in *Nrf2* and *GPX1* genes expressions. Our findings indicated that *Macleaya cordata* IA addition attenuated LPS-induced intestinal epithelium injury and disorder of intestinal homeostasis by enhancing the anti-inflammatory and antioxidant capacity of broiler chickens possibly via co-regulating TLR4/MyD88/NF-κB and Nrf2 signaling pathways.

## Introduction

The healthy gut is crucial for the maintaining homeostasis and defending against the invasive pathogenic microorganisms (1). Nevertheless, with the continuous development of the intensive breeding mode, the risk of intestinal damage in broilers also increases because of the effects of pathogenic bacteria, variable environment, and feed mycotoxin contamination (2, 3). Bacterial infection-induced intestinal injury is particularly common in modern poultry production, characterized by chronic inflammatory response and oxidative stress (4). Nutritional regulation has been proved to be a successful approach for safeguarding the intestine (5). With a growing demand for safe and high-quality meat and meat products, looking for green and natural additives to address intestinal damage has become a hot spot in broiler production in recent years (6).

*Macleaya cordata* is a kind of medicinal plantbelonging to the *Papaveraceae* family. Its extract has been found that its extract has anti-tumor, anti-inflammatory, and antibacterial properties, making it an effective alternative to antibiotic growth promoter (7, 8). *Macleaya cordata* extract (MCE) contains abundant active ingredients, such as sanguinarine, chelerythrine, protopine, and allocryptopine (9). Compounds encompassing sanguinarine and chelerythrine have been officially approved as dietary supplements in the EU in 2005, and are widely used in animal production (10). However, the current literature evaluating the effects of *Macleaya cordata* isoquinoline alkaloids (IA) in animal production is limited. Recently, a veterinary drug named Bopu powder was registered in China (Veterinary Drug No. 180415374), consisting of IA isolated from *Macleaya cordata*, which might be used to cure *Escherichia coli* (*E. coli*)-induced chicken diarrhea. According to reports, dietary supplementation with IA improves the antioxidant capacity and promotes an increased abundance of advantageous microorganisms in the small intestine of laying hens (11). In addition, the dietary addition of 40 mg/kg Bopu powder with IA significantly affects intestinal development and function, as well as relieves intestinal inflammation and oxidative stress in broilers (12). However, it is still unclear whether the addition of *Macleaya cordata* IA to broiler diets can effectively mitigate the intestinal injury in broilers.

Lipopolysaccharide (LPS) is the main component of the Gram-negative bacterial cell wall, and is a common model for establishing intestinal injury in broiler chickens (13). Multiple studies have shown that LPS attack can adversely affect the permeability and structural integrity of intestinal cells in broiler chickens (14). Besides, LPS can upregulate the related gene expression of the Toll-like receptor 4 (TLR4)/myeloid differentiation factor 88 (MyD88)/nuclear factor kappaB (NF-κB) signaling pathway, then leading to inflammatory and oxidative damage in the intestines (15). To the best of our current knowledge, however, there is a limited amount of research studying on adding *Macleaya cordata* IA to alleviate LPS challenge-induced intestine injury in broilers. Therefore, the purpose of the present study was to further investigate the protective function of dietary *Macleaya cordata* IA supplementation on LPS-induced intestinal injury in broilers, and sought to further elucidate the mechanisms by which *Macleaya cordata* IA alleviated intestinal injury during LPS challenge in broilers.

## Materials and methods

### Animals, treatments, and feeding management

A total of 486 one-day-old Arbor Acres broiler chicks, each weighing an average of 48.76 ± 0.25 g, were procured from a nearby commercial hatchery. All broilers were selected and randomly allocated to one of the three treatments (six replicates per treatment with twenty-seven broilers in each replicate) as follows (1): control (CON) group, broilers were fed a basal diet (2); LPS group, LPS-challenged broilers were fed a basal diet (3); LPS+IA group, LPS-challenged broilers were fed a basal diet supplemented with 0.6 mg/kg *Macleaya cordata* IA. The active ingredient of IA was used in this study was protopine and allotypotopine (containing at least 0.4 mg/kg protopine and 0.2 mg/kg allotypotopine). The formulation of the components and nutritional levels in the basal diet (**Supplementary Table S1**) was in compliance with the standards set by the Ministry of Agriculture of China in 2004. The IA was obtained from Micolta Bioresource Company Limited (Changsha 410331, China) and premixed with starch before supplementation. The LPS (*E. coli* L2880; Sigma-Aldrich, MO, USA) was solubilized in 0.9% sterile saline solution. On days 17, 19, and 21, the LPS and LPS+IA groups were received intraperitoneal injections of LPS at a dose of 1 mg/kg body weight (16). On the other hand, the CON group was received an intraperitoneal injection of saline solution at the same volume. All the broiler chickens were housed within a three-level cage situated within an environmentally-controlled room that maintained a constant level of illumination. The controlled room maintained the necessary ventilation and temperature required for broilers. In addition, all broiler chickens in this investigation were immunized according to the normal vaccination protocol.

### Sample collection

After fasting for 12 hours on day 21, a random selection was made to select one broiler chicken from each replicate for the purpose of sampling. Blood samples were extracted from wing veins, placed in coagulation-promoting vacutainer tubes, and centrifuged at 3,000 *g* for 15 minutes at 4°C. The serum was preserved at −20°C. Broilers were euthanized after blood samples were obtained. In a sterile setting, the digestive tract was subsequently removed from each chicken. The segments of approximately 2 cm long were excised from the central portion of the small intestine and subjected to multiple flushes with 9% saline solution, and subsequently preserved in a 4% paraformaldehyde solution for histological analysis. Subsequently, mucosal tissue from the small intestine was collected by gently scraping the same parts with a sterile glass slide. The mucosal tissues were then promptly preserved at a temperature of −80°C. Additionally, the contents of the cecal were aseptically collected and also preserved at a temperature of −80°C for future microbiological analysis.

### Measurement of serum inflammatory cytokines concentrations

The serum levels of tumor necrosis factor-α (TNF-α), interleukin-1 (IL-1β), interleukin-6 (IL-6), and interleukin-10 (IL-10) in broilers were determined using ELISA kits (Jiangsu Meimian Industrial Co., Ltd.). This determination procedure was conducted following the methodology established in previous work conducted by Chen et al. (17).

### Measurement of serum DAO and D-lactate levels

The concentrations of diamine oxidase (DAO) and D-lactate in the serum were measured using ELISA kits (Jiangsu Meimian Industrial Co., Ltd.) following a previous protocol (12).

### Measurement of antioxidant parameters concentrations in serum and intestinal mucosa

Intestinal mucosal samples were precisely weighed and homogenized (1:9, wt/vol) ice-cold sterile saline solution. Next, the mixture was centrifuged at 12,000 × *g* for 15 min at 4°C to obtain the supernatant. The supernatant was stored at −20°C for further analysis of the related indicators. The levels of malondialdehyde (MDA), superoxide dismutase (SOD), total antioxidant capacity (T-AOC), hydrogen peroxide (H_2_O_2_), and glutathione (GSH) were determined in each serum and intestinal homogenate by commercial kits (Nanjing Jiancheng Bioengineering Institute) in accordance with the manufacturer’s instructions.

### Measurement of intestinal mucosal SIgA, inflammatory cytokines, and mucosal barrier parameters

The levels of secretory immunoglobulin A (SIgA), inflammatory cytokines (TNF-α, IL-1β, IL-6, IL-10, and IFN-γ), and mucosal barrier parameters [trefoil factor family member (TFF), transforming growth factor-α (TGF-α), mucin 2 (MUC2), and zonula occluden-1 (ZO-1)] in the broiler intestinal mucosa was measured by commercial ELISA kits purchased from Jiangsu Meimian Industrial Co., Ltd., following the protocols described in a prior study (12).

### Measurement of intestinal mucosal caspases activities

Caspase-3, caspase-8 and caspase-9 activity in the intestinal mucosa was determined using ELISA kits (Beyotime Biotechnology, Shanghai, China). Briefly, 10 mg of intestinal mucosa samples was homogenized using 100 μL of lysis buffer with a glass homogenizer, and then placed on an ice bath for 5 min. The mucosal supernatants were obtained after centrifugation at 20,000 *g* for 10 min. Then 100 μL of reaction mixture containing 40 μL of buffer solution, 50 μL of supernatant, and 20 μL of pre-cooling substrates of caspase-3, caspase-8, and caspase-9 (Ac-DEVD-pNA, Ac-IETD-pNA, and Ac-LEHD-pNA, respectively) were added into the plate wells, followed by incubation at 37 °C for 60 to 120 min. The measurement of absorbance of the mixtures was performed at 405 nm. The activity of intestinal mucosal caspase-8, caspase-9, and caspase-3 was standardized to each sample’s total protein concentration, which was determined using the Bradford Protein Assay Kit from Beyotime Biotechnology.

### Observation of intestinal morphology

The small intestine tissues were extracted from the 4% paraformaldehyde solution following a 24-hour fixing period. Subsequently, the tissues were embedded using the paraffin embedding process described in Zhang et al. (18). The tissue was sliced automatically (Burton International Trading Co., Ltd., China, HM355S) into 5µm-thick sections. Afterwards, hematoxylin-eosin staining was performed, and the small intestinal morphology was visualized and photographed according to the method previously described (17). For each sample, 6 well-oriented villus height (VH) and crypt depth (CD) were measured by an image analyzer (Lucia software, zadrahau), and the VH/CD ratio was calculated.

### Intestinal TUNEL assay

The terminal deoxynucleotidyl transferase-mediated deoxyuridine triphosphate nick-end labeling (TUNEL) test was employed to measure intestinal apoptosis as previously described (16). Firstly, the paraffin section of the broiler intestine was deparaffinized and rehydrated. Subsequently, after being dried and incubated with Proteinase K for 22 min in a 37°C incubator, the intestinal sections were washed three times, each for 5 min on a decolorization shaker. Excess liquid was then removed, and permeable working solution was gently poured onto the tissue until it was submerged before incubation at room temperature for 20 min. Next, dried and buffered samples were added, followed by the addition of the necessary quantities of TdT enzyme and dUTP. Subsequently, sections were placed flat in a wet box and incubated at 37°C for 2 h before being dried and washed, and then incubated in the dark with DAPI dye for 10 min. Finally, after washing and drying, the slides were sealed in order to observe apoptosis.

### Analysis of gene expression in the intestinal mucosa

The 50-100 mg intestinal tissue samples were ground, and total RNA was extracted from each sample using a 1 ml TRIzol-reagent RNA kit in line with the manufacturer’s specifications (Accurate Biotechnology Co., Ltd., China). Subsequently, according to the methodology outlined by Liu et al. (8), the RNA was reverse transcribed into cDNA using the reverse transcription (RT) kit, and qRT-PCR was performed using qPCR kit and gene specific primers. The primers shown in **Supplementary Table S2** were used to perform real-time quantitative PCR. The 2^-ΔΔCt^ method was used to calculate the relative expression levels of mRNA (8).

### Cecum microbiological analysis

Microbial genomic DNA from the cecal content of broiler chickens was extracted using QIAamp DNA·Stool Mini kits (Qiagen Inc., Hilden, Germany) in accordance with the manufacturer’s instructions. Following the determination of concentration and purity, the V4 hypervariable region of the 16S ribosomal DNA genes were amplified according to the method in Chen et al. (19). The assessment of the library quality was conducted using the Qubit@2.0 fluorometer (Thermo Scientific) and the Agilent Bioanalyzer 2100 system. Following that, the libraries that were produced underwent sequencing using the Illumina NovaSeq platform. Finally, the paired-end reads were processed through sequence assembly, data filtering, and the removal of chimeras to obtain the final sequences. Paired-end reads were merged using FLASH (v1.2.7). Under specific filtering conditions, the quality of the raw reads was filtered to acquire clean tags, high-quality tags in accordance with QIIME (v1.9.1). Chimeric sequences were identified by comparing them with the Silva database using the UCHIME algorithm. The resulting chimeric sequences were then subjected to quality filtering to extract the effective sequences from the raw tags. Using the Uparse software, sequences with at least 97% similarity were assigned to the same OTU, and the taxonomic information was annotated using the Silva Database on the Mothur algorithm (20, 21). Alpha diversity was assessed using the Shannon, Simpson, Chao1, and ACE indices, and visualized using R software (Version 2.15.3) (22). Beta diversity was calculated using QIIME and was based on the Bray-Curtis distance. A principal coordinate analysis was plotted to visualize uniformity distances (23). The significant differences of microbial communities were detected by the ANOSIM test.

### Statistical analysis

Data were analyzed using the SAS 9.4 software (SAS Inst. Inc., Carry, NC, USA). Analysis of variance was performed using Tukey’s multiple range test, and the experimental data were shown as the mean ± standard error (SE). Differences were considered statistically significant when *P < 0.05, **P < 0.01, and ***P < 0.001, and the ^#^0.05 ≤ *P* < 0.10 was considered a significant trend.

## Results

### Serum inflammatory factors levels

**Figure 1:**
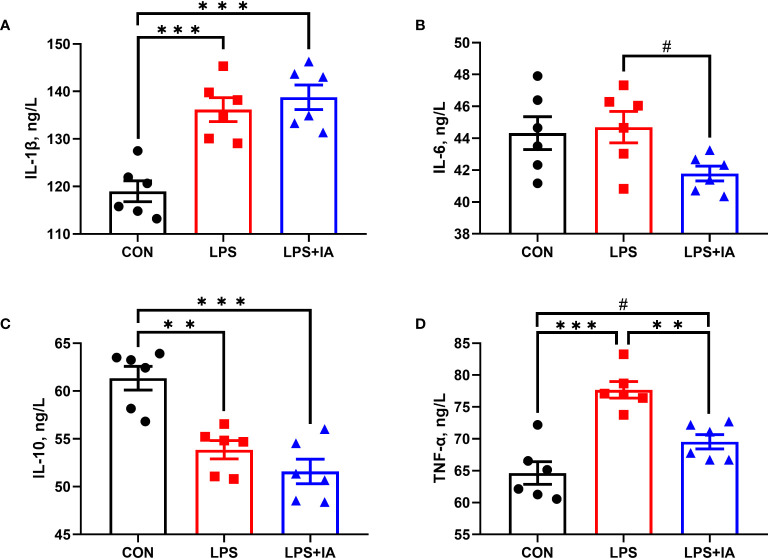
Effects of dietary supplementation with isoquinoline alkaloids on serum inflammatory in LPS-challenged broiler chickens. **(A)** Interleukin-1β (IL-1β); **(B)** Interleukin-6 (IL-6); **(C)** Interleukin-10 (IL-10); and **(D)** Tumor necrosis factor-α (TNF-α). CON group, broiler chickens fed a basal diet; LPS group, LPS-challenged broilers fed basal diets; and LPS+IA group, LPS-challenged broilers fed basal diets with isoquinoline alkaloids 0.6mg/kg. Values are presented as mean and standard error of mean (*n* = 6). #0.05 ≤ *P* < 0.10, **P < 0.01, ***P < 0.001.

Serum inflammatory factors levels in broilers are presented in **Figure 1**. The concentrations of serum IL-1β were notably lower in the CON group than those in the LPS and LPS+IA groups (*P* < 0.05), while the concentrations of serum IL-6 in the LPS+IA group tended to decrease in comparison to the LPS group (*P* < 0.10). Serum IL-10 concentrations were notably higher in the CON group compared with the LPS and LPS+IA groups (*P* < 0.05). LPS-challenged broilers exhibited a marked increase in the serum TNF-α concentration (*P* < 0.05) when compared with the CON and LPS+IA groups. The levels of serum TNF-α in the CON group demonstrated the tendency to decrease when compared with the group that received LPS and IA treatment (*P* < 0.10).

### Serum DAO and D-lactate concentrations

**Figure 2:**
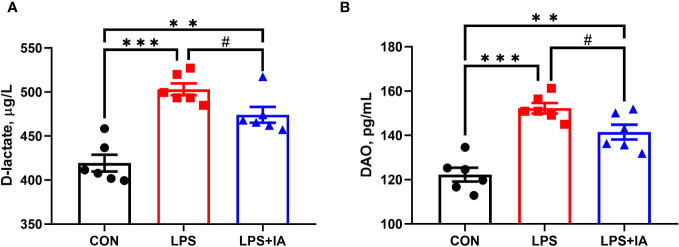
Effects of dietary supplementation with isoquinoline alkaloids on serum D-lactate and DAO in LPS-challenged broiler chickens. **(A)** DAO; and **(B)** D-lactate. CON group, broiler chickens fed a basal diet; LPS group, LPS-challenged broilers fed basal diets; and LPS+IA group, LPS-challenged broilers fed basal diets with isoquinoline alkaloids 0.6mg/kg. Values are presented as mean and standard error of mean (*n* = 6). #0.05 ≤ *P* < 0.10, **P < 0.01, ***P < 0.001.

**Figure 2** shows that serum D-lactate and DAO concentrations were dramatically lower in the CON group in comparison to the LPS and LPS+IA groups (*P* < 0.05). Additionally, the LPS+IA group’s serum D-lactate and DAO concentrations tended to be decreased in comparison to than those of the LPS group (*P* < 0.10).

### Intestinal morphology and pathology

**Figure 3:**
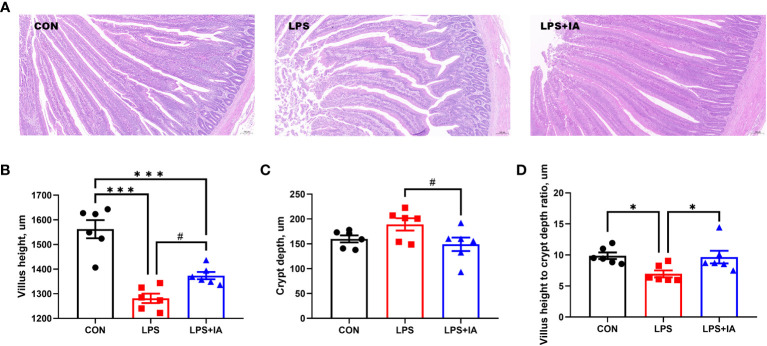
Effects of dietary supplementation with isoquinoline alkaloids on intestinal morphology and pathology in LPS-challenged broiler chickens. **(A)** Representative images (×100) were stained with hematoxylin and eosin; **(B)** Villus height; **(C)** Crypt depth; and **(D)** Villus height to crypt depth ratio. CON group, broiler chickens fed a basal diet; LPS group, LPS-challenged broilers fed basal diets; and LPS+IA group, LPS-challenged broilers fed basal diets with isoquinoline alkaloids 0.6mg/kg. Values are presented as mean and standard error of mean (*n* = 6). #0.05 ≤ *P* < 0.10, *P < 0.05, ***P < 0.001.

Intestinal histopathological micrographs are shown in the **Figure 3A**. Compared to the LPS group, the intestinal villus structure of the CON and LPS+IA groups were clear and complete, with almost no damage. However, in the LPS group, the integrity of the intestinal villi was compromised, leading to the infiltration of a significant number of inflammatory cells.

Intestinal morphology was measured as depicted in **Figures 3B–D**. In comparison to the LPS and LPS+IA groups, the small intestine from the CON group exhibited significantly increased villus height (*P* < 0.05). Meanwhile, compared to the CON and LPS+IA groups, the VH to CD ratio was notably decreased in the LPS group (*P* < 0.05). Additionally, compared with LPS group, the intestinal VH in the LPS+IA group tended to increase and the intestinal CD tended to decrease (*P* < 0.10).

### Serum and intestinal mucosal antioxidant indexes

**Figure 4:**
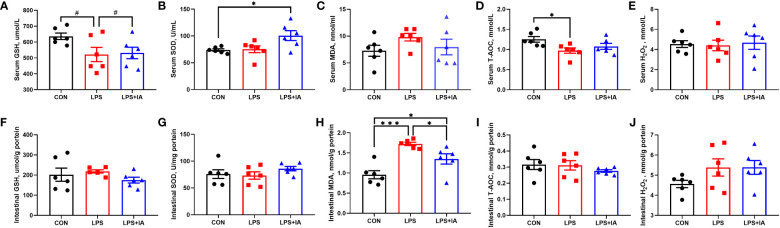
Effects of dietary supplementation with isoquinoline alkaloids on serum and intestinal antioxidant indexes in LPS-challenged broiler chickens. **(A, F)** Glutathione, (GSH); **(B, G)** Superoxide dismutase, (SOD); **(C, H)** Malondialdehyde, (MDA); **(D, I)** Total antioxidant capacity, (T-AOC); and **(E, J)** Hydrogen peroxide, (H_2_O_2_). CON group, broiler chickens fed a basal diet; LPS group, LPS-challenged broilers fed basal diets; and LPS+IA group, LPS-challenged broilers fed basal diets with isoquinoline alkaloids 0.6mg/kg. Values are presented as mean and standard error of mean (*n* = 6). #0.05 ≤ *P* < 0.10, *P < 0.05, ***P < 0.001.

The serum and intestinal antioxidant indexes are depicted in **Figure 4**. The concentration of serum SOD in the LPS+IA group was notably elevated (*P* < 0.05) and the levels of serum T-AOC in the LPS group were notably decreased (*P* < 0.05) when compared to the CON group. Besides, the levels of intestinal mucosal MDA in the LPS group were notably increased compared with both the CON and LPS+IA groups (*P* < 0.05). Additionally, the amount of intestinal mucosal MDA was notably elevated in the LPS+IA group compared to the CON group (*P* < 0.05). Compared to the CON and LPS+IA groups, the serum levels of GSH in the LPS group showed a tendency to decline (*P* < 0.10). Nevertheless, no differences in the serum concentrations of MDA and H_2_O_2_, intestinal mucosal SOD, T-AOC, H_2_O_2_, and GSH among the three groups were observed (*P* > 0.05).

### Intestinal mucosal barrier function

**Figure 5:**
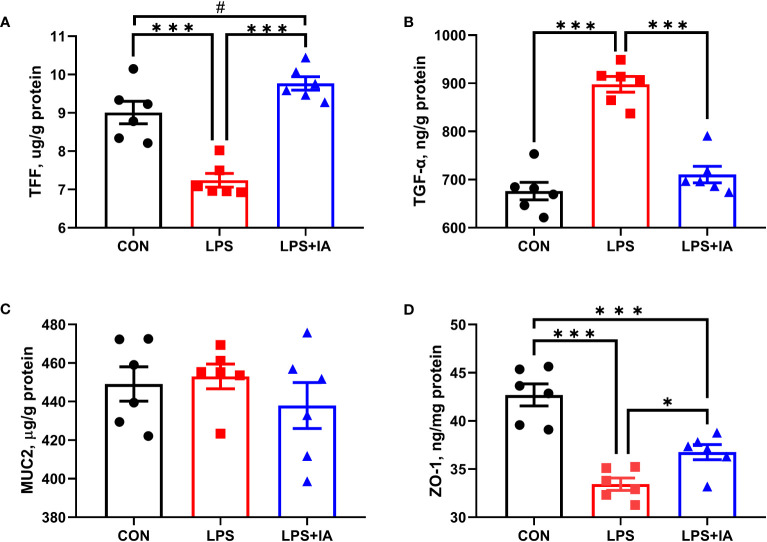
Effects of dietary supplementation with isoquinoline alkaloids on intestinal barrier function in LPS-challenged broiler chickens. **(A)** Trefoil peptides (TFF); **(B)** Transforming growth factor alpha (TGF-α); **(C)** Mucin 2 (MUC2); and **(D)** Zonula occludens-1 (ZO-1). CON group, broiler chickens fed a basal diet; LPS group, LPS-challenged broilers fed basal diets; and LPS+IA group, LPS-challenged broilers fed basal diets with isoquinoline alkaloids 0.6mg/kg. Values are presented as mean and standard error of mean (*n* = 6). #0.05 ≤ *P* < 0.10, *P < 0.05, ***P < 0.001.

As depicted in **Figure 5**, a significant decrease in intestinal mucosal TFF and ZO-1 concentrations was shown in both the CON and LPS+IA groups compared to the LPS group (*P* < 0.05). Furthermore, the concentration of intestinal mucosal ZO-1 in the LPS+IA group was significantly lower than that in the CON group (*P* < 0.05). The levels of intestinal mucosal TFF in the LPS group exhibited a tendency towards elevation in comparison to the CON group (*P* < 0.10). Nevertheless, no significant interactions in the concentration of MUC2 in intestinal mucosa among the three groups were observed (*P* > 0.05).

### Intestinal mucosal apoptosis indicators

#### Intestinal mucosal caspase activity

**Figure 6:**
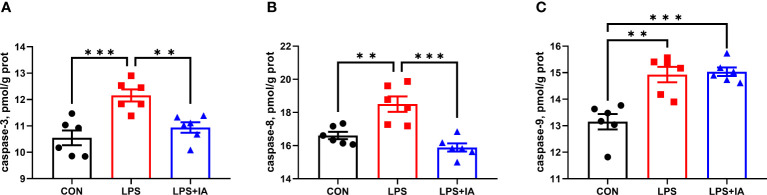
Effects of dietary supplementation with isoquinoline alkaloids on caspases levels in LPS-challenged broiler chickens. **(A)** caspase-3; **(B)** caspase-8; and **(C)** caspase-9. CON group, broiler chickens fed a basal diet; LPS group, LPS-challenged broilers fed basal diets; and LPS+IA group, LPS-challenged broilers fed basal diets with isoquinoline alkaloids 0.6mg/kg. Values are presented as mean and standard error of mean (*n* = 6). **P < 0.01, ***P < 0.001.

**Figure 6** illustrates the outcomes of intestinal mucosal caspase activities. Compared to the CON group, LPS-challenged broilers had a markedly elevated intestinal mucosal activity of caspase-3, caspase-8, and caspase-9 (*P* < 0.05). Compared with the broilers of LPS group, dietary IA addition significantly decreased the intestinal mucosal activity of caspase-3 and caspase-8 (*P* < 0.05). Intestinal mucosal caspase-9 activity was downregulated in CON broilers compared to LPS+IA broilers (*P* < 0.05).

#### Intestinal apoptotic index

**Figure 7:**
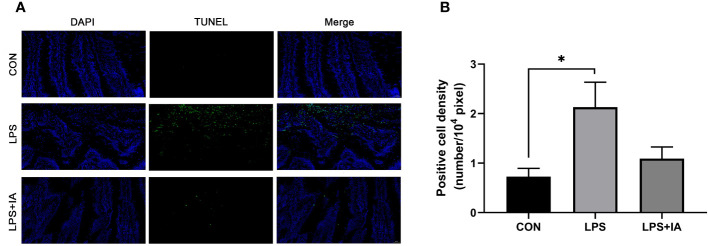
Effects of dietary supplementation with isoquinoline alkaloids on intestinal apoptotic index in LPS-challenged broiler chickens. **(A)** TUNEL assay. The blue color represents the total cells, and the green color represents the apoptosis cells. **(B)** Positive cell density. CON group, broiler chickens fed a basal diet; LPS group, LPS-challenged broilers fed basal diets; and LPS+IA group, LPS-challenged broilers fed basal diets with isoquinoline alkaloids 0.6mg/kg. Values are presented as mean and standard error of mean (*n* = 6). *P < 0.05.

The intestinal apoptotic index measured by the TUNEL assay is depicted in **Figure 7**. **Figure 7A**, the green fluorescence of the LPS group was significantly greater than in the CON and LPS+IA groups. However, there was little green fluorescence in the CON and LPS+IA groups, indicating that there was a greater proportion of apoptotic cells in the intestine of the LPS group. In addition, in **Figure 7B**, the density of TUNEL-positive cells in the LPS group was notably increased compared to the CON group (*P* < 0.05). Nevertheless, no significant difference found in the density of TUNEL-positive between LPS+IA and other groups (*P* > 0.05).

#### Intestinal mucosal inflammatory factors and intestinal mucosal SIgA concentrations

**Figure 8:**
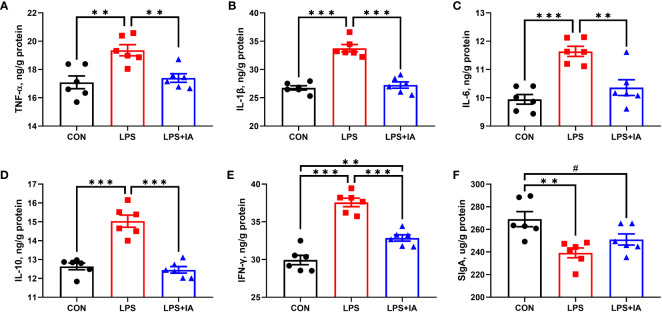
Effects of dietary supplementation with isoquinoline alkaloids on intestinal inflammatory factors and intestinal SIgA concentrations in LPS-challenged broiler chickens. **(A)** Tumor necrosis factor-α (TNF-α); **(B)** Interleukin-1β (IL-1β); **(C)** Interleukin-6 (IL-6); **(D)** Interleukin-10 (IL-10); **(E)** Interferon-γ (IFN-γ); and **(F)** Secretory immunoglobulin A (SIgA). CON group, broiler chickens fed a basal diet; LPS group, LPS-challenged broilers fed basal diets; and LPS+IA group, LPS-challenged broilers fed basal diets with isoquinoline alkaloids 0.6mg/kg. Values are presented as mean and standard error of mean (*n* = 6). #0.05 ≤ *P* < 0.10, **P < 0.01, ***P < 0.001.

As represented in **Figure 8**, concentrations of TNF-α, IL-1β, IL-6, IL-10, and IFN-γ were considerably higher in the LPS broilers compared with CON and LPS+IA broilers (*P* < 0.05). The intestinal mucosal concentration of IFN-γ in the LPS+IA group was significantly higher than that of the CON group (*P* < 0.05). Additionally, the intestinal mucosal SIgA concentration in CON broilers was markedly higher than that of the LPS group (*P* < 0.05), and it tended to be higher than that of LPS+IA broilers (*P* < 0.10).

#### Intestinal gene expressions

**Figure 9:**
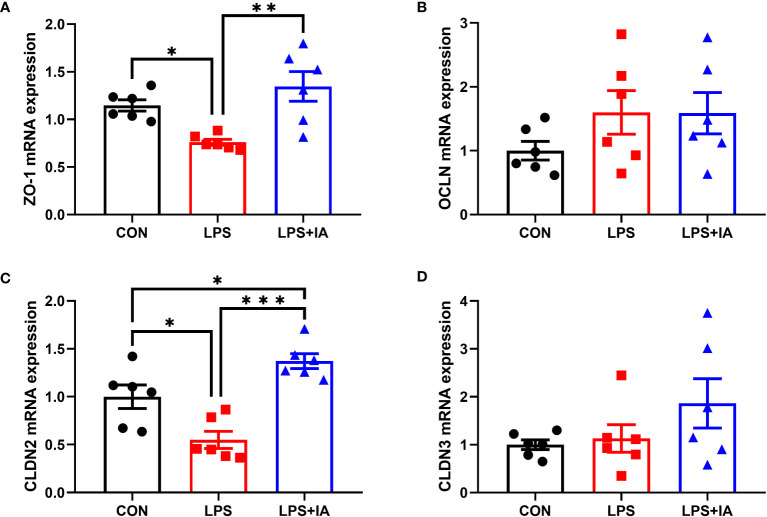
Effects of dietary supplementation with isoquinoline alkaloids on the expression of barrier genes in LPS-challenged broiler chickens. **(A)** Zonula occludens-1 (*ZO-1*); **(B)** Occludin (*OCLN*); **(C)** Claudin-2 (*CLDN2*); and **(D)** Claudin-3 (*CLDN3*). CON group, broiler chickens fed a basal diet; LPS group, LPS-challenged broilers fed basal diets; and LPS+IA group, LPS-challenged broilers fed basal diets with isoquinoline alkaloids 0.6mg/kg. Values are presented as mean and standard error of mean (*n* = 6). *P < 0.05, **P < 0.01, ***P < 0.001.

Intestinal barrier function related gene expression is displayed in **Figure 9**. The LPS group displayed a significant reduction in the expression of *ZO-1* and claudin-2 (*CLDN2*) compared to both the CON and LPS+IA groups (*P* < 0.05). Additionally, the LPS+IA group displayed markedly increased *CLDN2* mRNA expression compared to the CON group (*P* < 0.05). No discernible disparities in the relative gene expression of occludin (*OCLN*) and claudin-3 (*CLDN3*) were observed among the three groups (*P* > 0.05).

**Figure 10:**
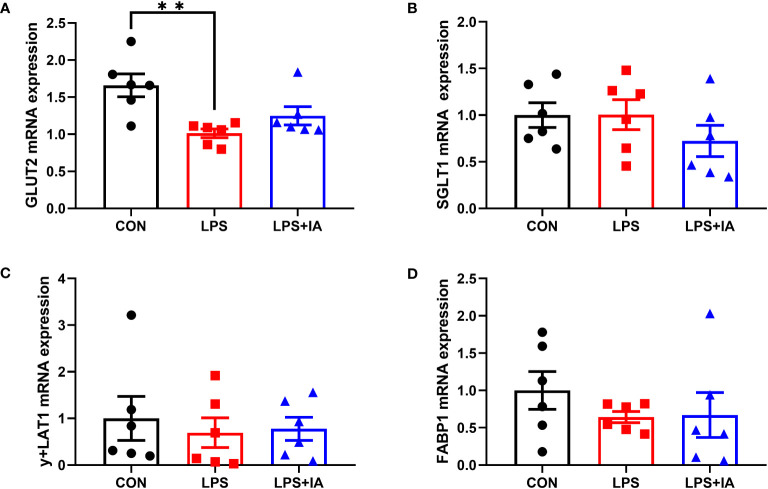
Effects of dietary supplementation with isoquinoline alkaloids on the expression of nutrient transport genes in LPS-challenged broiler chickens. **(A)** Glucose transporter type 2 (*GLUT2*); **(B)** Sodium-glucose transporter 1 (*SGLT1*); **(C)** y + L amino acid transporter-1 (*y + LAT1*); and **(E)** Fatty acid binding protein-1 (*FABP1*). CON group, broiler chickens fed a basal diet; LPS group, LPS-challenged broilers fed basal diets; and LPS+IA group, LPS-challenged broilers fed basal diets with isoquinoline alkaloids 0.6mg/kg. Values are presented as mean and standard error of mean (*n* = 6). **P < 0.01.

Gene expression related to intestinal nutrient transporters is displayed in **Figure 10**, expression of glucose transporter type 2 (*GLUT2*) was significantly inhibited among the LPS group compared to the CON group (*P* < 0.05). Nevertheless, no notable differences in the mRNA expression of sodium-glucose transporter 1 (*SGLT1*), y+L amino acid transporter-1 (*y+LAT1*), and fatty acid binding protein-1 (*FABP1*, *P* > 0.05) were found among the three groups.

**Figure 11:**
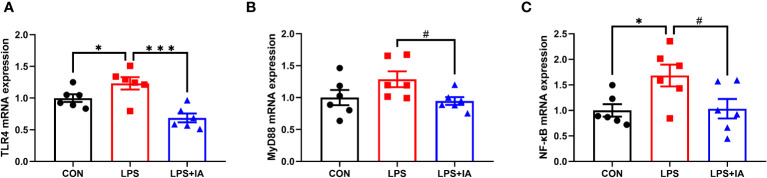
Effects of dietary supplementation with isoquinoline alkaloids on the expression of inflammatory genes in LPS-challenged broiler chickens. **(A)** Toll-like receptor 4 (*TLR4*); **(B)** Myeloid differentiation primary response 88 (*MyD88*); and **(C)** Nuclear factor-kappa B (*NF-κB*). CON group, broiler chickens fed a basal diet; LPS group, LPS-challenged broilers fed basal diets; and LPS+IA group, LPS-challenged broilers fed basal diets with isoquinoline alkaloids 0.6mg/kg. Values are presented as mean and standard error of mean (*n* = 6). #0.05 ≤ *P* < 0.10, *P < 0.05, ***P < 0.001.

As shown in **Figure 11**. Expression of intestinal *TLR4* mRNA in the LPS group was markedly elevated in comparison to the CON and LPS+IA groups (*P* < 0.05). Besides, compared with to the LPS group, the *NF-κB* mRNA expression was notably decreased in the CON group (*P* < 0.05), while *MyD88* and *NF-κB* mRNA expression tended to decrease in the LPS+IA group (*P* < 0.10).

**Figure 12:**
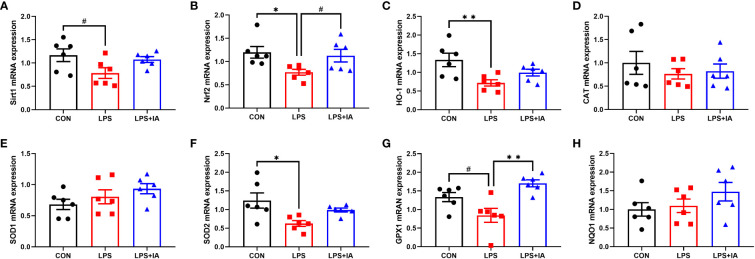
Effects of dietary supplementation with isoquinoline alkaloids on the expression of antioxidant genes in LPS-challenged broiler chickens. **(A)** Sirtuin1 (*Sirt1*); **(B)** Nuclear factor erythroid 2-related factor 2 (*Nrf2*); **(C)** Heme-oxygenase 1 (*HO-1*); **(D)** Catalase (*CAT*); **(E)** Superoxide dismutase 1 (*SOD1*); **(F)** Superoxide dismutase 2 (*SOD2*); **(G)** glutathione peroxidase-1 (*GPX1*); and **(H)** NAD(P)H quinone oxidoreductase 1 (*NQO1*). CON group, broiler chickens fed a basal diet; LPS group, LPS-challenged broilers fed basal diets; and LPS+IA group, LPS-challenged broilers fed basal diets with isoquinoline alkaloids 0.6mg/kg. Values are presented as mean and standard error of mean (*n* = 6). #0.05 ≤ *P* < 0.10, *P < 0.05, **P < 0.01.

Gene expression related to intestinal antioxidant capacity is displayed in **Figure 12**. In comparison to the CON group, the expression of *Nrf2*, heme-oxygenase 1 (*HO-1*), and superoxide dismutase 2 (*SOD2*) in the LPS group showed markedly reduced expression (*P* < 0.05), while the mRNA expression of sirtuin1 (*Sirt1*) and glutathione peroxidase-1 (*GPX1*) tended to decrease in the LPS group (*P* < 0.10). Dietary supplementation with IA led to a marked increase in the expression of the *GPX1* (*P* < 0.05), and tended to increase *Nrf2* expression (*P* < 0.10) compared with LPS-challenged broiler chickens. Nonetheless, no differences observed among the groups in terms of the expression of catalase (*CAT*), superoxide dismutase 1 (*SOD1*), and NAD(P)H quinone oxidoreductase 1 (*NQO1*, *P* > 0.05).

**Figure 13:**
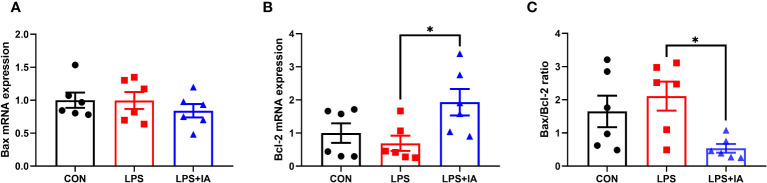
Effects of dietary supplementation with isoquinoline alkaloids on the expression of apoptosis genes in LPS-challenged broiler chickens. **(A)** Bcl-2 associated X (*Bax*); **(B)** B-cell lymphoma-2 (*Bcl-2*); and **(C)** Bax/Bcl-2 ratio. CON group, broiler chickens fed a basal diet; LPS group, LPS-challenged broilers fed basal diets; and LPS+IA group, LPS-challenged broilers fed basal diets with isoquinoline alkaloids 0.6mg/kg. Values are presented as mean and standard error of mean (*n* = 6). *P < 0.05.

Intestinal apoptosis-related gene expression is displayed in **Figure 13**. In comparison to the LPS group, dietary IA addition significantly increased the expression of b-cell lymphoma-2 (*Bcl-2*) and decreased the *Bax/Bcl-2* ratio (*P* < 0.05). Additionally, no difference in the bcl-2 associated X (*Bax*) expression was found among the groups (*P* > 0.05).

### Cecum microbiological composition and diversity

#### Microbial diversity of cecum microorganisms

**Figure 14:**
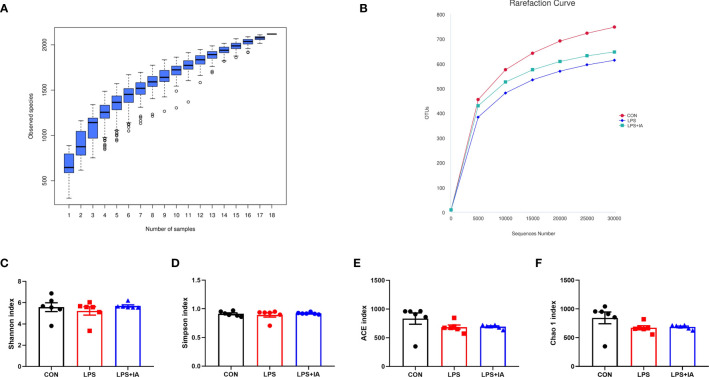
Alpha diversity and richness of the cecal microbiota. **(A)** The species accumulation curves; **(B)** The rarefaction curve of OTU; **(C)** Shannon index; **(D)** Simpson index; **(E)** ACE index; and **(F)** Chao 1 index. CON group, broiler chickens fed a basal diet; LPS group, LPS-challenged broilers fed basal diets; and LPS+IA group, LPS-challenged broilers fed basal diets with isoquinoline alkaloids 0.6mg/kg. Values are presented as mean and standard error of mean (*n* = 6).

The results depicted in **Figures 14A, B** demonstrate that as the number of sequences analyzed increased to 18, the species accumulation curve exhibited a tendency to plateau. Additionally, the rarefaction curve approached the asymptote, suggesting that the number of samples analyzed was sufficient for conducting the OTU test and predicting species richness within the samples. The alpha diversity was shown in **Figures 14C–F**, the Shannon, Simpson, Chao1, and ACE were not statistically significant among all groups (*P* > 0.05).

**Figure 15:**
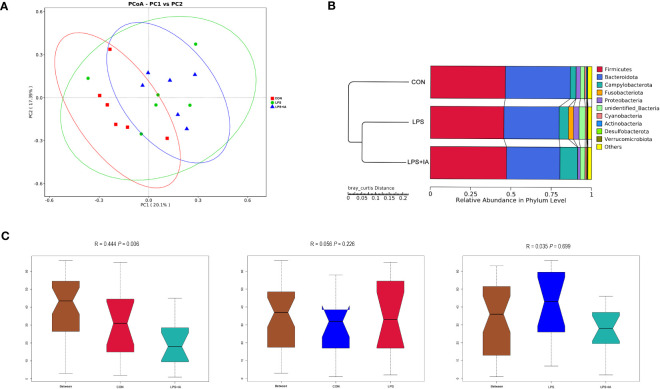
Beta diversity analysis of the cecal microbiota. **(A)** The principal coordinate analysis (PCoA); **(B)** Unweighted pair-group method with arithmetic mean (UPGMA) phylogenetic tree; and **(C)** The analysis of ANOSIM. CON group, broiler chickens fed a basal diet; LPS group, LPS-challenged broilers fed basal diets; and LPS+IA group, LPS-challenged broilers fed basal diets with isoquinoline alkaloids 0.6mg/kg. *n* = 6. Differences between groups were considered significant at *P* < 0.05.

Also, the principal coordinate analysis confirmed that the LPS and LPS+IA groups were far apart from the CON group, indicating that LPS injection caused significant changes in the bacterial composition of the cecal microbiota (**Figure 15A**). In this study, the UPGMA phylogenetic tree (**Figure 15B**) revealed a close association between the LPS and LPS+IA groups, as the two groups clustered together with the highest level of similarity. Conversely, the CON group exhibited a distinct distribution pattern, occupying a separate branch. This observation implies that there is a significant difference in the distribution of the CON group compared to the other groups. According to the findings presented in **Figure 15C**, the ANOSIM analysis demonstrated that no significant difference in the composition of microbial community between the CON and LPS groups, as well as between the LPS and LPS+IA groups (*P* > 0.05). However, the CON and LPS+IA groups showed a significant dissimilarity in microbial community composition (*P* < 0.05).

#### Relative abundance of cecal microorganisms

To identify significant differences in the taxonomic composition of the cecal microorganisms among the CON, LPS, and LPS+IA groups, LEfSe analysis was conducted (**Supplementary Figure S1**). The findings indicated that the LPS group had a significantly increased relative abundance of the phyla Fusobacteriota and Verrucomicrobiota, along with genera *Ligilactobacillus*, *Fusobacterium*, *Barnesiella*, and *Enterococcus*. Besides, the LPS+IA group showed a significantly increased relative abundance of genera *Megamonas*, *UCG-008*, and *Streptococcus*. Finally, the CON group exhibited a significantly higher relative abundance of the genus *Sphingomonas*.

## Discussion

The intestinal barrier functions as the initial protective barrier against detrimental microorganisms, playing a vital role in maintaining the stability of intestinal homeostasis and preventing the invasion of pathogenic microorganisms (1). The intestinal barrier comprises a mechanical barrier, chemical barrier, immune barrier, and microbial barrier, and each barrier is closely related and interacts with each other to resist the invasion of harmful pathogens (24). Maintaining the integrity of intestinal epithelial morphology is crucial for maintaining mechanical barrier function (25). Our current study revealed that the injection of LPS resulted notable pathological alterations in the gastrointestinal tract of broiler chickens. The results indicated that there was morphological damage of the intestinal villi and inflammation responses. This indicates that the model effectively led to impaired intestinal function, aligning with the prior discoveries made by Lv et al. (13). Moreover, increased villus height and an increased ratio of VH to CD are indicative of enhanced efficiency in nutrient absorption across the intestinal barrier (17). Our research showed that the broilers receiving both LPS challenge and *Macleaya cordata* IA exhibited a notably elevated VH to CD ratio compared to the broilers receiving only LPS challenge. These results suggest that adding *Macleaya cordata* IA may help protect the structure of the crypt-villus from damage caused by LPS.

The intestinal mechanical barrier is not only the key for the body to resist harmful pathogens from the outside world, but also the foundation for maintaining intestinal mucosal epithelial permeability and barrier function (24). Our study’s findings indicate that the LPS infection led to increased levels of DAO and D-lactate in the blood. However, adding *Macleaya cordata* IA exhibited a reduction in broiler serum levels of DAO and D-lactate. Meanwhile, the levels of ZO-1 and TFF, along with the expression of *ZO-1*, *CLDN2*, and *GLUT2* in the broiler intestines were significantly reduced upon LPS administration. DAO and D-lactate acted as markers for assessing intestinal permeability. It was found that the DAO and D-lactate levels will be notably increased when the intestine was damaged (26). Closely linked genes such as ZO-1, OCLN, CLDN2, and CLDN3 have a key role in intestinal barrier function and cell permeability (27). Previous work has shown that elevated peripheral mucosal ZO-1 levels contribute to the regulation of intestinal integrity and barrier function (28). Trefoil peptides were elevated to help prevent intestinal disease and maintain the intestinal barrier (29). In our experiment, supplementation with *Macleaya cordata* IA increased the levels of intestinal mucosal ZO-1 and TFF, as well as intestinal mucosa of *ZO-1* and *CLDN2* mRNA levels, in LPS-challenged broiler chickens. Therefore, our results suggested that adding IA to the broiler diet might protect intestinal integrity and barrier function from LPS challenge by regulating the expression of tight junction proteins. Interestingly, our data also revealed significantly higher levels of intestinal TGF-α in the LPS group. The high TGF-α levels can promote the maintenance of intestinal epithelial cell integrity (30). However, excessive expression of TGF-α can also trigger the onset of inflammation (31).

The intestinal immune barrier is mainly composed of immune cells, immune factors, and intestinal-related lymphatic tissues (32). SIgA is the predominant immunoglobulin found in intestinal secretions and is crucial for the intestinal immune system’s function. SIgA is primarily synthesized by plasma cells, which can prevent pathogens from adhering to the intestinal epithelium, thereby enhancing the immune barrier function of the intestinal epithelium (33). In our current study, we observed that the concentration of intestinal SIgA decreased after LPS challenge. However, when *Macleaya cordata* IA was added, the concentration of intestinal SIgA in these broilers increased. This suggests that the supplementation of *Macleaya cordata* IA might enhance immune function and potentially alleviate LPS-induced injury to the intestinal epithelium.

It is well established that the microbiota in the gut mainly plays the role of a biological barrier by preventing the colonization of the body by bacteria that are harmful (34). Because of this, it is recognized that changes in its composition have a significant role in the development of various diseases. Studies have reported that the LPS challenge can threaten the equilibrium of the gut microbiota, ultimately resulting in the development of gut dysbiosis (35). Between the CON and LPS+IA groups, we found significant changes in microbial community composition, suggesting that *Macleaya cordata* IA supplementation could change the abundance of microbial communities, which was congruent with one of our earlier studies (12). In the LPS group, the LPS challenge resulted in an increased abundance of some potentially harmful microorganisms, such as Fusobacteriota, *Fusobacterium*, *Barnesiella*, *Akkermansia, and Enterococcus.* Numerous studies have indicated a significant association between *Fusobacterium*, *Enterococcus*, and various diseases, including multibacterial sepsis and chronic inflammatory bowel disease (36, 37). Additionally, increased *Barnesiella* levels have also been shown to correlate with decreased body weight and increased levels of inflammatory factors (38). Meanwhile, the increased *Akkermansia* levels have also been demonstrated to be closely related to inflammation (39). Interestingly, the LPS challenge has also been found to result in an increased abundance of beneficial microorganisms, such as Verrucomicrobiota and *Ligilactobacillus*. A recent study found that Verrucomicrobiota was specific bacterial phylum in the gut of patients with cerebral intraparenchymal hemorrhage, and a similar enrichment of Verrucomicrobiota has been found in the gut of mice with traumatic brain injury (40, 41). Another study showed that pathogens associated with calf diarrhea cause indigestion and malabsorption, increasing the amount of glucose and galactose entering the distal small intestine and colon, and indirectly leading to excessive growth of lactate-producing bacteria (42). In our study, the LPS challenge resulted in a decrease in the intestinal mRNA expression of GLUT2, which may lead to the increase in intestinal glucose levels. Therefore, we believe that the increase in lactobacillus abundance may be due to LPS-induced intestinal glucose metabolism disorder. Furthermore, our current study demonstrated that dietary supplementation of LPS-challenged broilers with *Macleaya cordata* IA increased the abundance of the genera *Megamonas*, *UCG-008*, and *Streptococcus*. *Megamonas* has been shown to be effective in inhibiting LPS-induced intestinal inflammatory damage and promoting intestinal health (43). The *UCG-008* is a member of the *Butyricicoccaceae* family and is closely associated with butyrate production (44). *Streptococcus* are commensal organisms and potential probiotics in chickens, which can reduce pathogen colonization (45). However, among the CON group, the heightened abundance of *Sphingomonas* might cause intestinal inflammation (46). These results indicated that LPS challenge led to pathogen invasion and intestinal barrier damage, while *Macleaya cordata* IA supplementation could resist pathogen colonization and invasion by maintaining a consistent microbial community composition.

Excessive apoptosis of epithelial cells has been a major cause of intestinal mucosal barrier damage (17). Preserving a balance between cell apoptosis and proliferation performs a crucial role in controlling the turnover of the intestinal epithelium (47). In the current study, we used the TUNEL method to find more positive cell staining in the intestinal mucosa of LPS group. Meanwhile, the LPS-challenge increased the *Bax* expression, as well as the activity of caspase, and decreased the expression of *Bcl-2* in the intestinal mucosa of broiler chickens. Previous studies have demonstrated that caspase-8 and caspase-9 could cause apoptosis and promote cell death by activating caspase-3 (48, 49). Additionally, the regulation of apoptosis is dependent on maintaining a delicate balance between Bcl-2 and Bax. It has been established that caspase-3 activation is associated with an increased Bax/Bcl-2 ratio (50, 51). Our current results demonstrated that dietary supplementation with IA decreased intestinal mucosal activity of caspase-3, caspase-8 and the *Bax/Bcl-2* ratio, while increasing intestinal mucosal *Bcl-2* expression in broilers, which was generally consistent with our previous findings (12). Hence, our results suggest that supplementation with *Macleaya cordata* IA in the broiler diet could reduce intestinal apoptosis through the inhibition of capase-3 activity, thereby alleviating LPS injection-induced intestinal injury.

Elevated concentrations of inflammatory factors could lead to inflammation in the body, disturbance of the intestinal barrier, and increased intestinal permeability (52, 53). Numerous investigations have revealed that LPS could cause intestinal inflammation by increasing the levels of intestinal mucosal pro-inflammatory cytokine (16, 54). In this study, LPS injection increased the serum and intestinal TNF-α, IL-1β, and IL-6 concentrations, and intestinal IFN-γ concentrations. However, research has found that supplementing *Macleaya cordata* IA can restore the levels of pro-inflammatory factors in the serum and intestinal mucosa of broiler chickens. This indicates that *Macleaya cordata* IA can effectively reduce the production of pro-inflammatory factors. Significantly, the addition of *Macleaya cordata* IA to the broiler diet was shown to significantly reduce the intestinal mucosa and serum of IL-10 concentrations. The decreased concentrations of IL-10 might be associated to the diminished presence of anti-inflammatory cytokines (55). To identify possible mechanisms of supplementation with *Macleaya cordata* IA in alleviating LPS-induced intestinal epithelium damage, we quantified the gene expression levels pertaining to the TLR4/MyD88/NF-κB pathway in the intestinal mucosa. Multiple studies have shown that lipopolysaccharide (LPS) could stimulate the synthesis of inflammatory cytokines by activating the TLR4/MyD88/NF-κB signalling pathway (56, 57). Previous reports indicate that the TLR4 receptor system could identify LPS molecules and trigger the downstream protein MyD88, which in turn activates the NF-κB signaling pathway, leading to the initiation of an inflammatory response (58). In our study, LPS injection increased the mRNA expression levels of the intestinal TLR4/MyD88/NF-κB pathway-related gene in broiler chickens. However, dietary supplementation with IA was able to alleviate the TLR4/MyD88/NF-κB pathway-related gene mRNA expressions in broiler chickens’ intestine mucosa. Similarly, dietary supplementation with IA inhibited the TLR4/MyD88/NF-κB signaling pathway and significantly decreased levels of intestinal inflammatory factors in a previous study (12). Inhibition of TLR4/MyD88/NF-κB signaling pathway could inhibit inflammatory damage and protect the intestine (59). Therefore, our findings suggest that supplementation with *Macleaya cordata* IA can attenuate LPS-induced intestinal inflammation damage by modulating the TLR4/MyD88/NF-κB signaling pathway.

The generation of oxidative stress usually accompanies the inflammatory response as well (60). In enteric diseases, oxidative stress is a key cause in the disruption of the intestinal barrier. Malondialdehyde is closely linked to oxidative damage and serves as one of the final products of lipid peroxidation, which can hence be used to assess the degree of lipid peroxidation (61, 62). Moreover, the antioxidant defense system primarily consists of various antioxidant factors, such as T-AOC, SOD, and GSH. The increased levels of antioxidant factors indicate an enhanced ability of the body to scavenge free radicals (63, 64). Besides, the Sirt1/Nrf2 signaling system is regarded as a prototypical antioxidant signaling pathway for regulating oxidative stress. Activation of Sirt1 has been found to protect epithelial cells of the intestine from oxidative injury by regulating Nrf2-related pathways (65). Nrf2 is a critical multifunctional regulator factor in oxidative stress, and Nrf2 activation could upregulate the expression of downstream antioxidant-related targets against oxidative stress (66). Previous reports indicated that in intestinal epithelial cells, activating the Nrf2 signaling pathway could effectively inhibit the production of reactive oxygen species, enhance the survival of cells, and improve the redox states of cells (67). The HO-1, SOD, and GPX1 are all crucial antioxidative enzymes that play distinct roles in regulating cellular reactive oxygen species levels. Nrf2 could regulate the level of reactive oxygen species (ROS) in cells by activating HO-1 to suppress the production of ROS and protect against oxidative stress (66). Meanwhile, the activation of Nrf2 could also upregulate the expression of SOD and improve the level of free radical removal (63). GPX1 is a stress-type glutathione peroxidase and has a crucial function in reducing and removing oxidative stress (68). In our experiments, we observed that injecting LPS significantly increased the intestinal MDA levels while decreasing serum T-AOC and levels of mRNA for *Sirt1*, *Nrf2*, *HO-1*, *SOD2*, and *GPX1* in the broiler intestines. However, supplementation of IA in the broiler diet could significantly decrease intestinal MDA levels, increase serum SOD levels and intestinal *GPX1* expression, and alleviate the decrease in the expression of Sirt1/Nrf2 antioxidant signaling pathway-related gene. Similarly, our previously study had shown that supplementation with *Macleaya cordata* IA could enhance antioxidant capacity in broilers via activation of the Nrf2 pathway (12). Hence, our results indicate that supplementation with *Macleaya cordata* IA could alleviate intestinal oxidative damage, and its mechanism may be achieved through the activation of the Nrf2 signaling pathway.

## Conclusion

Collectively, we reveal that supplementation with 0.6 mg/kg *Macleaya cordata* IA in the broiler diet can overcome LPS-induced intestinal mucosal damage by enhancing anti-inflammatory and antioxidant capacity, which may be related to the inhibition of the TLR4/MyD88/NF-κB signaling pathway and the activation of the Nrf2 signaling pathway. Our research results confirm the immunomodulatory and anti-inflammatory properties of *Macleaya cordata* IA as well as its ability to exert an antioxidant effect and alter the gut microbiota. Therefore, *Macleaya cordata* IA shows promise as a valuable feed supplement to prevent intestinal injury in broiler chickens.

## Data availability statement

Publicly available datasets were analyzed in this study. This data can be found here: All sequencing data are deposited in the Sequence Read Archive of the National Center for Biotechnology Information under accession PRJNA1003399 (Illumina sequences).

## Ethics statement

The animal study was reviewed and approved by the Care and Use Committee of Shandong Agricultural University (Ethics Approval code: SDAUA-2021-019). The study was conducted in accordance with the local legislation and institutional requirements.

## Author contributions

YLiu: Conceptualization, Data curation, Formal analysis, Methodology, Supervision, Visualization, Writing – original draft. KH: Data curation, Formal analysis, Project administration, Writing – original draft. HL: Conceptualization, Resources, Writing – review & editing. GJ: Resources, Software, Writing – review & editing. LC: Methodology, Writing – review & editing. GW: Data curation, Methodology, Writing – review & editing. ZP: Data curation, Methodology, Writing – review & editing. YZ: Data curation, Methodology, Writing – review & editing. SJ: Investigation, Resources, Software, Writing – review & editing. NJ: Conceptualization, Investigation, Resources, Software, Writing – review & editing. LH: Investigation, Software, Supervision, Validation, Visualization, Writing – review & editing. WY: Conceptualization, Funding acquisition, Writing – review & editing. YLi: Conceptualization, Funding acquisition, Software, Validation, Writing – review & editing.
