# Supplementary material for: Macleaya cordata isoquinoline alkaloids attenuate Escherichia coli lipopolysaccharide-induced intestinal epithelium injury in broiler chickens by co-regulating the TLR4/MyD88/NF-κB and Nrf2 signaling pathways
Source: Front Immunol. 2024 Jan 17;14:1335359. doi: 10.3389/fimmu.2023.1335359 (PMC10828024; doi:10.3389/fimmu.2023.1335359)
Supplement: Supplementary file 1 [file DataSheet_1.pdf]

Supplementary Material

Supplementary Figures and Tables

Table S1. Composition and nutrient levels of basal diets (as-fed basis)

Items	Phases
Ingredients，%	
Corn	55.91
Soybean meal, 44% CP	13.78
Wheat bran	11.98
Corn starch residue	7.99
Corn gluten meal	3.99
Extruded soybean	1.50
Limestone	1.70
Calcium monophosphate	1.10
L-Lysine HCl	1.00
DL-Methionine	0.20
L-Threonine	0.10
Sodium chloride	0.40
Choline	0.10
Phytase	0.10
Complex enzyme	0.02
Trace mineral premix [1]	0.10
Vitamin premix [2]	0.02
Antioxidant	0.02
Total	100
Calculated analysis，%	
Metabolizable energy，MJ/kg	12.33
Crude protein	19.47

Crude fat	3.45
Calcium，%	0.94
Available phosphorus，%	0.35
Lysine，%	1.15
Methionine，%	0.50

[1] Provided per kilogram of complete basal diet: 10 mg of Cu as $CuSO_4$, 100 mg of Fe as $FeSO_4$, 1.1 mg of I as $Ca(IO_3)_2$, 65 mg of Zn as $ZnSO_4$, 100 mg of Mn as $MnSO_4$ and 0.3 mg of Se as Na_2SeO_3.

[2] Provided per kilogram of complete basal diet: vitamin A 10,000 IU, vitamin D_3 3,000 IU, vitamin E 30 IU, vitamin K_3 1.3 mg, vitamin B_1 2.2 mg, vitamin B_2 8 mg, vitamin B_3 8 mg, vitamin B_6 4 mg, vitamin B_{12} 0.025 mg, biotin 0.2 mg, niacin 40 mg, folic acid 1 mg and D-calcium pantothenate 10 mg.

Table S2. Primer sequences used for quantitative real-time PCR.

Genes[b]	GenBank	Primer sequences, 5′-3′[a]	Size, bp
β-actin	NM_205518.1	F:ATTGTCCACCGCAAATGCTTC<br>R:AAATAAAGCCATGCCAATCTCGTC	113
ZO-1	XM_015278981.2	F:CCACCTCAGAATAAGCCAGCAAT<br>R:CGGTTGTAAGAAGGAGTGACTGTT	146
OCLN	NM_205128.1	F:ATCAACGACCGCCTCAATCAG<br>R:TCCTCTGCCACATCCTGGTATT	83
CLDN2	NM_001277622.1	F:ACATTGGTTCAAGCATCGTGAC<br>R:GCTGTAGATGTCGCACTGAGT	101
CLDN3	NM_204202.1	F:GCCAAGATCACCATCGTCTCC<br>R:ATCACCAGCGGGTTGTAGAAAT	116
GLUT2	NM_207178.2	F:TTTCGAGAGAGCCGGTGTTG<br>R:GCCTTCTCCACCAGGAAGAC	102
SGLT1	NM_001293240.1	F:CATCGTTATCCTGGCAGTCTCCTT<br>R:TCATCGGGTTTCTCCTCCTCATCA	138
y+LAT1	XM_418326.5	F:CTCTCTCTCATCATCTGGGC<br>R: TCATTCCTGGGTCTGTTGCT	472
CAT1	NM_001145490	F:CTCTGGCTTGGTGGTGAACATCT<br>R:CGTGCTTGGCTTGAGGGTAGT	88
FABP1	NM_204192.4	F:ACTGGCTCCAAAGTAATGACCAATG<br>R:TGTCTCCGTTGAGTTCGGTCAC	132
TLR4	NM_001030693.2	F:CATCTCTGGAGTTCCTGCTGAA<br>R:TGTATGGATGTGGCACCTTGA	145
MyD88	NM_001030962.5	F:CGGAGGATGGTGGTCGTCATT<br>R:TCGTTCTTCATGGTCTTGCACTTG	140
NF-κB	NM_001396038.1	F:CAGCCCATCTATGACAACCG<br>R:TCAGCCCAGAAACGAACCTC	152
Sirt1	NM_001004767.1	F:CACGCCTTGCTGTAGACTTCC<br>R:ATGAACTTGTGGCAGAGAGATGG	148
Nrf2	MN416129.1	F:CACGCCTTGCTGTAGACTTCC<br>R:ATGAACTTGTGGCAGAGAGATGG	109
HO-1	NM_205344.2	F:GTCCCGAATGAATGCCCTTGA<br>R:ATGACCGTTCTCCTGGCTCTT	139
CAT	NM_001031215.2	F: GGAGGTAGAACAGATGGCGTATG<br>R: CGATGTCTATGCGTGTCAGGAT	114
SOD1	NM_205064.2	F:CGCAGGTGCTCACTTCAATCC<br>R:CAGTCACATTGCCGAGGTCAC	89
SOD2	NM_204211.2	F:GCTGTATCAGTTGGTGTTCAAGGA<br>R:GCAATGGAATGAGACCTGTTGTTC	130
GPX1	NM_001277853.3	F:CGGCTTCAAACCCAACTTCAC<br>R:CTCTCTCAGGAAGGCGAACAG	85

NQO1	NM_001277619.2	F:GAGTGCTTTGTCTACGAGATGGA<br>R:ATCAGGTCAGCCGCTTCAATC	104
Bax	XM_422067	F:TGAGCATGTAGCAACGGAAG<br>R:AGCAAGCTGATTGACGGTCT	295
Bcl-2	NM_205339.3	F:AGGACAACGGAGGATGGGATG<br>R:CACCAGAACCAGGCTCAGGAT	110

[a] F: forward primer; R: reverse primer

[b] *ZO-1*, zonula occludens-1; *OCLN*, occludin; *CLDN2*, claudin-2; *CLDN3*, claudin-3; *GLUT2*, glucose transporter type 2; *SGLT1*, sodium-glucose transporter 1; *y+LAT1*, y+L amino acid transporter-1; *CAT*, catalase; *FABP1*, fatty acid binding protein-1; *TLR4*, toll-like receptor 4; *MyD88*, myeloid differentiation primary response 88; *NF-κB*, nuclear factor-kappa B; *Sirt1*, sirtuin1; *Nrf2*, nuclear factor erythroid 2-related factor 2; *HO-1*, heme-oxygenase 1; *SOD1*, superoxide dismutase 1; *SOD2*, superoxide dismutase 2; *CAT*, catalase; *GPX1*, glutathione peroxidase-1; *NQO1*, NAD(P)H quinone oxidoreductase 1; *Bax*, bcl-2 associated X; *Bcl-2*, b-cell lymphoma-2.

Supplementary Figures

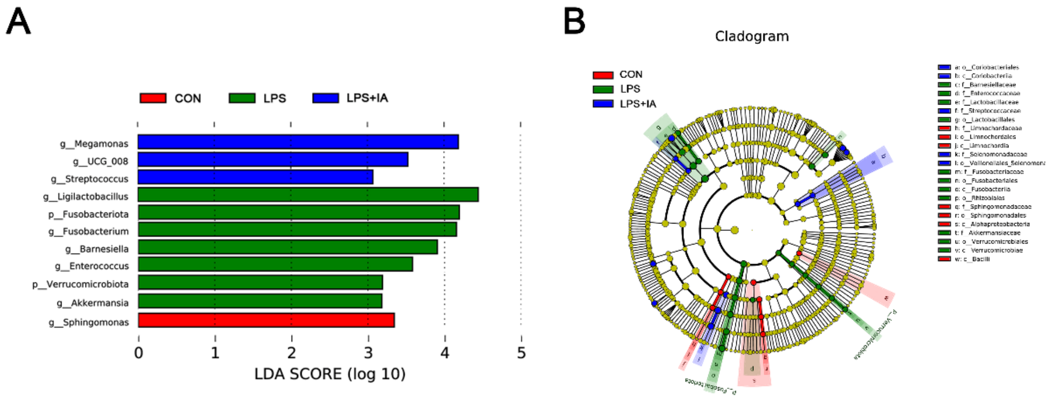

Figure S1. LEfSe analysis of the Cecal microbiota. (A) The log-transformed LDA scores of bacterial taxa identified through LEfSe analysis. Only taxa meeting an LDA significant threshold >3 are shown. (B) The taxonomic cladogram of bacterial taxa obtained through LEfSe analysis.
